# Field Study of Postural Characteristics of Standing and Seated Smartphone Use

**DOI:** 10.3390/ijerph19084583

**Published:** 2022-04-11

**Authors:** Yi-Lang Chen, Kuan-Hsiang Chen, Yu-Cheng Cheng, Chien-Ching Chang

**Affiliations:** Department of Industrial Engineering and Management, Ming Chi University of Technology, New Taipei 24301, Taiwan; u07217034@mail2.mcut.edu.tw (K.-H.C.); u07217048@mail2.mcut.edu.tw (Y.-C.C.); u07217901@mail2.mcut.edu.tw (C.-C.C.)

**Keywords:** smartphone user, neck flexion, head flexion, standing and sitting, backrest

## Abstract

Prior investigations have been primarily conducted in a laboratory to examine the effects of the smartphone use on the neck and head positions, whether these results are applicable to actual conditions is still unknown. This field survey thus analyzed the neck flexion (NF), head flexion (HF), gaze angle (GA), and viewing distance (VD) of smartphone users in public areas in Taipei, Taiwan. Six hundred smartphone users (300 men and 300 women) were photographed sagittally in standing, supported sitting, or unsupported sitting postures while using a smartphone. Results showed that women had significantly less NF and HF and shorter VDs than male users. Regardless of gender, higher NF was observed for standing than for sitting. Women had similar NF and HF while sitting supported and unsupported, but both were significantly lower than those while standing. By contrast, male users had higher NF and HF during unsupported sitting than during supported sitting. The NF (45°–50°) was much greater than the recommended maximum safe NF of 15°. Women may be at higher risk of visual strain because of shorter VD.

## 1. Introduction

Smartphones have become the primary tool for information acquisition, messaging, and interpersonal interaction in daily life [1]. In 2016, approximately 34% of that year’s global population owned a smartphone, and this number has gone considerably up to 3.8 billion smartphone users, which means that currently over 48% of the world’s population own a smartphone [2]. Such proportions near or exceed 90% in many regions, such as South Korea, Japan, Taiwan, and Southeast Asia [3], and in the USA and Australia, smartphone ownership is even higher among adults, with 92% and 95% of the adults aged 18 to 34 years respectively reported to own a smartphone [4]. Smartphone users are no longer a minority group but the preponderant majority of the population [5]. In restaurants, in lines, or on the train, whether standing or sitting, people today keep their smartphones on hand and constantly use them to connect with the outside world.

Many physical and psychological problems result from smartphone use. Such problems place strain on the human neck and shoulder region and the prospective associations were found between smartphone use and the follow-up neck/shoulder symptoms [6]. Korhan and Elghomati [7] reviewed relevant literature published from 2010 to 2019 and concluded that the most common self-reported discomfort is that in the neck and shoulders, and the prevalence of discomfort is 32.5–85.6%. Park et al. [8] found that smartphone use induced more flexed posture on the neck and trunk than other visual display terminal work and also changed posture and muscle activation within a relatively short amount of time (5 min); neck and shoulders pain after 16 min of smartphone use was reported. Namwongsa et al. [9] also discovered that the neck was the most painful body region after the use of smartphones over a 12-month period, and one of the factors associated with neck disorders was a flexed neck posture, with an odds ratio of 2.44.

As mentioned, Eitivipart et al. [10] conducted a systematic survey of effect of smartphone use on musculoskeletal disorders and pain and indicated that the neck-related muscle activations were increased as well as head flexion angle and forward head shifting which increased during the smartphone use. Generally, posture-related strain on the neck region can be quantified by measuring the head and neck forward flexion (HF and NF, respectively) in the sagittal plane. An observational study of mobile device usage conducted by Gold et al. [11] found that almost all participants had a flexed neck (91.0%, *n* = 782). Han et al. [12] used wearable motion sensors to collect data on HF in college students during 8 h of typical smartphone use and discovered that smartphone users tended to spend more time with HF exceeding 30° during smartphone use than during other activities. Szeto et al. [13] also reported significantly greater cervical and upper thoracic flexion during smartphone use than during nonuse. These results indicate that smartphone use and high NF are correlated. Lee et al. [14] reported that HF was significantly greater when participants were texting or sitting than when they were browsing or standing. HF was affected by different smartphone operations and user postures.

Straker et al. [15] indicated that increased NF causes increased activation of the neck and shoulder muscles, which stabilize the head position and maintain neck balance. Thus, NF increases the loads on the cervical erector spinae (CES) and upper trapezius (UT) muscles [16]. However, Namwongsa et al. [17] found that when the neck was gradually flexed forward, CES activation increased, whereas UT activation decreased. For NF of 0°–15°, activation of both the CES and UT was acceptably low, suggesting that NF < 15° during smartphone use could prevent discomfort. Recently, Tapanya et al. [18] found that the lowest gravitational moment of the neck at 0° flexion was associated with the lowest CES activation and the lowest neck discomfort scores. They concluded that maintaining 0° NF during standing texting could alleviate smartphone-related neck discomfort and that excessive NF (30° and 45° flexion) could exacerbate discomfort. However, smartphone users usually have forward HF of approximately 33°–45° [14]. Ning et al. [19] also observed that people may have increased NF (44.7°) during smartphone use. Higher NF causes more strain on the cervical spine. For an adult with a neutral head position, 44–53 N of force is applied to the cervical spine, but this force increases to 120 N when the neck is flexed 15°, to 178 N at 30°, to 218 N at 45°, and to 267 N at 60° [20]. This relationship is approximately linear.

The rapidly increasing utilization of smartphones makes ophthalmic problems associated with their use an important issue [21]. Viewing distance (VD) is another key metric of posture during smartphone use. Bababekova et al. [22] indicated that average VDs were 36.2 and 32.2 cm for reading messages and for Internet browsing on smartphones, respectively. These distances are both shorter than the typical VDs for other electronic devices [23] and much shorter than the 40 cm typical for reading physical documents [22]. Viewing a screen or target at a close range increases visual demands (e.g., accommodation and convergence) and may increase intraocular pressure [24] and exacerbate the symptoms of asthenopia.

Although neck and shoulder discomfort may be related to long-term smartphone use and to excessive HF and NF, Toh et al. indicated that the limited evidence for mobile touch screen device (MTSD, such as smartphones) use and the various aspects of its use are associated with musculoskeletal symptoms and exposures. This is due to mainly low quality experimental and case-control laboratory studies and further research is warranted to develop guidelines for wise use of MTSDs [25]. Korhan and Elghomati also indicated that the relevant studies are still insufficient and that no hazard assessment method can effectively determine what musculoskeletal diseases are induced by smartphone use. Therefore, it is useful to conduct further experimental studies related to the use of devices to address the potential complaints and reduce effects of using musculoskeletal disorders [7]. Prior investigations have been primarily conducted in a laboratory. Even though participants were requested to adopt a natural posture in these experiments, such methodology may produce Hawthorne (or observer) effects [26,27]. Therefore, in this study, 300 male and 300 female smartphone users were photographed while operating a smartphone and unaware of the photographer. Informed consent was later obtained. This study compared the HF, NF, VD, and gaze angle (GA) of users operating smartphones standing, sitting with a backrest, and sitting without a backrest, when they were in public areas. The results also compared with that obtained from the laboratory experiments of the previous studies.

## 2. Materials and Methods

### 2.1. Participants

A total of 600 smartphone users (300 men and 300 women) were randomly selected and successfully observed in public areas in Taipei, Taiwan, including university campuses, subway stations, MRT cars, lines, and parks. The data were collected between 20 June and 15 August 2021. The mean (standard deviation) age, height, and weight of the male users in this study were 33.5 (8.6) year, 170.6 (6.8) cm, and 63.1 (5.2) kg, respectively, and those of the female users were 31.7 (6.9) y, 159.2 (4.7) cm, and 56.3 (4.4) kg. All testing procedures were performed in accordance with the 2013 World Medical Association Declaration of Helsinki and were approved by the Research Ethics Committee of National Taiwan University in Taiwan (NTU-REC 202012-EM-25). All users provided written consent and their basic information (including smartphone size) if they agreed to participate in the study after they had been photographed; otherwise, the photos were immediately deleted. Informed consent obtained from all participants was also attested for publication of the identifying information/images in an online open-access publication.

### 2.2. Posture Measurement

**Figure 1:**
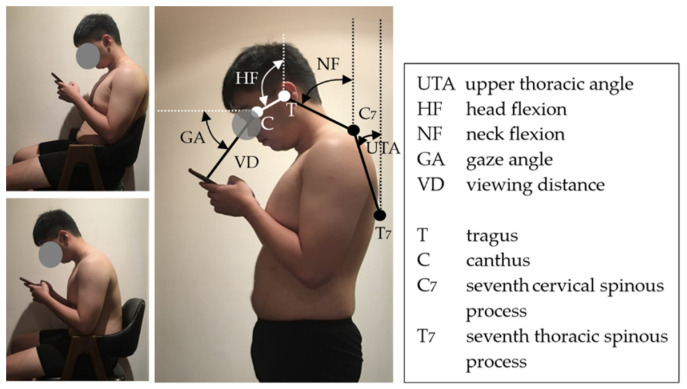
Schematic of anatomical markers and of measured angles and distance.

HF, NF, GA, and VD are visualized in Figure 1. By measuring symmetrical sagittal photos and employing CorelDRAW (Graphics Suite 2020, Corel Co., Ottawa, ON, Canada), the seventh cervical (C7) and thoracic (T7) spinous processes, canthus, and tragus and half of the length of the smartphone were marked on the photos digitally. The upper thoracic angle (UTA), NF, HF, GA, and VD were then calculated.

Because HF and NF are influenced by trunk position, researchers usually measure the trunk angle on the basis of a line from the acromial shelf to the hip [28,29]. However, this measurement method is substantially affected by the shoulder posture, lumbar flexion, and pelvic rotation [30,31]. Guan et al. [32] thus used the UTA for HF and NF measures. The UTA was defined as the angle between the line from C7 to T7 and a vertical line. This method is feasible because the upper thorax can be considered a rigid body. The experiment performed by Guan et al. [32] was conducted in a laboratory, and the UTA could thus be directly measured; in this field study, the participants were requested to stand normally and were then recorded for later UTA measurement after their postures of smartphone use had been photographed. This measurement was used to calibrate the HF and NF measurements. Yoon employed a similar method for NF measurements [5].

### 2.3. Measurement Validity and Reliability

To ensure the validity of the field measurement, we employed a camera (resolution = 1:30,000 in the camera field of view at 60 Hz, motion capture system, Qualisys MacReflex, Sweden) to validate the measurement by the CorelDRAW software adopted in the study. Ten participants (5 males and 5 females) wearing summer clothes, as we observed in the field study, were asked to sit and use their smartphones naturally, with or without body joint markers attached, respectively, and their postures were photographed with a motion analysis system. Images were then analyzed through MacReflex system for calculating joint angles and VD and CorelDRAW for identifying the joint positions and estimating the angles and VD by three experimenters. The data obtained from CorelDRAW were validated based on that from MacReflex system.

Three experimenters photographed user postures and subsequently recorded the measurement data. To ensure angles and distances were consistent among photos taken by different experimenters, before the experiment, an orthopedic physician with 15 years of clinical experience trained the experimenters to identify anatomical landmarks. Before data acquisition, the intraobserver and interobserver reliabilities were determined. Correlation coefficients (r) > 0.8 indicate satisfactory intraobserver and interobserver reliability. The mean absolute difference (MAD) was also used to examine differences in the estimated angles and distance for each observer or among the observers. Sixty samples were randomly selected from the 600 total samples (10 samples for each gender and posture combination), and the three experimenters conducted repeated measurements (with two measurement intervals of >6 h) for reliability analyses.

### 2.4. Study Design and Procedure

**Figure 2:**
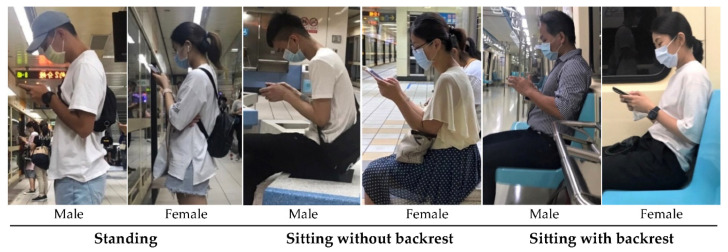
Three typical postures of smartphone use for both genders in the field study.

This study collected HF, NF, GA, and VD data for six smartphone use situations (two genders x three postures (standing, sitting with or without a backrest), as shown in Figure 2). The experimenters randomly sampled and photographed the smartphone users. A total of 600 smartphone users participated for the six combinations in the study; once 100 qualified samples were successfully collected for any combination, sampling of the combination was stopped. To ensure the consistency of the data, the field photography works were performed by experimenters through the same type of cameras (Sony, ZV-E10 + 16-50MM KIT, Tokyo, Japan). The camera was approximately aligned to the smartphone users’ shoulder height, and photographs were taken perpendicular to the user’s sagittal plane, and the maximum focal length of the camera was set to minimize the distortion. After the photographs were taken, the users were then asked if they were willing to participate in the study. Users giving consent then signed a consent form and provided basic personal information (i.e., age, stature, and body height). The experimenters also recorded the smartphone size and photographed the natural (habitual) standing posture when the participants held the smartphone for the normalization of VD and UTA measurements. If there was no object for normalization, the distance between participants’ tragus and canthus was then used with reference to the body height ratio. After photographing, if a user was unwilling to participate, the photographs were immediately deleted. Most users agreed to participate in the study. One reason for the high consent rate (79%) may be that people wear masks in public areas during the COVID-19 pandemic and have fewer privacy concerns. Outlier photographs, such as those of users carrying large backpacks, carrying heavier objects (>5% of their body weight [28]), with the smartphone placed on the legs, in abnormal sitting postures, or for whom body landmark identification was difficult, were excluded from the data. The unusual protocol of first taking a photograph and then requesting consent was necessary to avoid observation effects and capture natural posture during smartphone use. The CorelDRAW software was used to identify and mark the anatomical landmarks of the head, cervical spine, and thoracic spine on the photos (as shown in Figure 1) and then to measure angles and distances. These angle data were converted into net angles by subtracting the UTA, and subsequent analyses were performed.

### 2.5. Statistical Analysis

The data collected in the study were analyzed by using the SPSS 23.0 statistical software (IBM Corp., Armonk, NY, USA) with a significance level of α = 0.05 for all tests. The Pearson product–moment correlation (r) was used to explore the intraobserver and interobserver reliabilities for each dependent variable. Two-way analysis of variance (ANOVA) was used to compare the two genders and three postures for HF, NF, GA, and VD. The independent variables included gender and posture. One-way ANOVA for men and women was conducted if the interaction effect of 2-way ANONA was significant. Each participant was considered a block, and Duncan’s multiple range test was used for post hoc comparisons. Beforehand, the Kolmogorov–Smirnov test was used to verify the compliance of numerical variables with the normal distribution, while the Levene’s test was used to verify the homogeneity of variances.

## 3. Results

### 3.1. Measurement Validity and Reliability

Results of the validity test showed that differences in NF, HF, GA, and VD between two measurements (MacReflex system and data estimated manually by three experimenters through CorelDRAW) were 3.0°, 2.8°, 2.4°, and 1.4 cm, respectively, showing a satisfactory validity for the CorelDRAW software. The intraobserver and interobserver reliabilities for body landmark identification are displayed in Table 1, and these reliabilities for the angle and distance measurements are displayed in Table 2. The intraclass correlation coefficients for the four measurements were 0.952–0.993, demonstrating high internal consistency. The interclass correlation coefficient between any two observers was 0.934–0.993, indicating good reliabilities among the three experimenters.

### 3.2. Two-Way ANOVA Results

Through the Kolmogorov–Smirnov test, the data collected in the study were normally distributed meanwhile Levene’s test showed the data were homogenous (all *p* > 0.05). Table 3 displays the results of two-way ANOVA with gender and posture as the independent variables. Gender and posture significantly affected HF, NF (both *p* < 0.05), VD (*p* < 0.001), and GA (*p* < 0.001). The interaction of gender and posture significantly affected NF (*p* < 0.05) and GA (*p* < 0.05), despite power values of <0.8.

Duncan’s MRT results revealed that values of HF, NF, and VD for men were higher than those for women: 49.1° and 47.7° for HF, 97.6° and 94.7° for NF, and 36.7 and 31.3 cm for VD, respectively. As shown in Table 4, standing smartphone use caused the greatest HF (99.9°) and NF (51.7°); NF for sitting without a backrest (unsupported sitting; 49.1°) was lower than NF for standing but higher than NF for sitting with a backrest (supported sitting; 46.3°). No significant difference was found in HF between unsupported sitting (93.5°) and supported sitting (95.1°). The standing GA was 53.9°, which was significantly higher than the 41.2° and 44.4° observed for unsupported and supported sitting, respectively; differences between the seated positions were not significant.

### 3.3. One-Way ANOVA Results for Each Gender

The results of one-way ANOVA for each gender are displayed in Table 5. Posture significantly affected HF, NF, and GA (but not VD) for both genders (all *p* < 0.05). Duncan’s MRT results revealed differences between genders (Table 6). NF for both male and female users was greatest when standing and operating a smartphone; however, a significant difference was observed between genders for NF. When men sat unsupported, NF was 52.3°—nearly identical to that when standing (52.6°). When men sat supported, NF was 46.2°. Use of a backrest decreased average NF by 6.1° for sitting male smartphone users. Women had similar NF while sitting supported and unsupported, but both were significantly lower than those while standing.

## 4. Discussion

Unlike in laboratory experiments in which participants’ clothing can be controlled, anatomical markers are often concealed during field observation by street clothing, and posture is more difficult to determine. To ensure the consistency of the HF, NF, GA, and VD measurements, intraobserver and interobserver reliabilities were measured and determined to be satisfactory.

A total of 600 men and women were observed during smartphone use with different postures in public areas in Taipei. Smartphone users had the highest NF (51.7°) while standing; lower NF (49.1°) was observed for unsupported sitting, and users had the lowest NF (46.3°) during supported sitting. The greatest GA (53.9°) was observed of standing users, significantly greater than HF during unsupported (41.2°) or supported (44.4°) sitting. The difference in GA between the two sitting conditions was not significant. The results suggest a compensatory phenomenon between NF and GA for sitting postures. During supported sitting, NF tended to be lower, and GA tended to be higher. By contrast, NF was higher, and GA was lower during unsupported sitting. The results demonstrate that a backrest can reduce NF and thus alleviate the load on the extensor muscles of the cervical spine [17,18].

Namwongsa et al. [17] suggested that NF should be less than 15° when operating a smartphone to minimize cervical spine strain because CES activity is low at angles of 0°–15°. If NF exceeds 15°, CES activity increases substantially. However, the recommendation of no more than 15° of NF during smartphone use does not reflect natural way of using the smartphones in real life situations [33] and is substantially different from the NF of 33°–45° and 44.7° observed by Lee et al. [14] and Ning et al. [19], respectively. User NF was similarly observed to range from 45° to 50° for all postures in this study. Therefore, during typical smartphone use, NF far exceeds the 15° proposed by Namwongsa et al. [17] and the maximum safe NF angles of 30° and 45° suggested by Tapanya et al. [18]. However, differences in NF results among studies may be due to the different definitions of head/neck flexion, and most importantly, an exposed time duration for smartphone use is another crucial factor that causes neck and shoulder pain [9,34]. A survey of smartphone users in Taiwan (*n* = 1300) revealed that 51.5% of people use smartphones for 2–5 h daily and that 28.1% use smartphones for >5 h a day [35]. The relatively high exposure may increase the risk of pain.

Female smartphone users had significantly lower NF, HF, and VD than male users. Higher NF was measured for both genders standing than for sitting. Female users had similar NF and HF when sitting supported or unsupported, and both were significantly less during standing smartphone use. By contrast, when men operated a smartphone during unsupported sitting, higher NF and HF were observed than during supported sitting, but no difference was found for standing. Although women have a greater range of cervical motion [36], HF and NF in men were greater than those in women by 2.9° and 1.4°, respectively, consistent with the results of Guan et al. [32]. Greater NF combined with a smaller range of cervical motion implies that male users are at a higher risk for cervical spine injuries than female users are.

VD was approximately 34 cm for all postures. Boccardo [37] reported that average VDs were 37.4 and 36.1 cm for standing and sitting smartphone use, respectively. Although the difference in VD was only 1.3 cm, it was statistically significant. The average VD of nonpresbyopic participants was 35.0 cm—a similar result to ours. Boccardo [37] also indicated that the average VD as 34.7 cm for women and 38.2 cm for men; similar to this study, in which female users had shorter VDs than men. However, all the VD values obtained by Boccardo were higher than those in this study because that study investigated older adults and 43.7% of the participants had presbyopia. Shorter VDs during smartphone use are thought to cause more severe eye strain symptoms [38]. The VD of women in the study (average 31.3 cm) was significantly shorter than that of men (average 36.7 cm) and those reported in previous studies [22,37]; short VD may be a risk factor for women operating smartphones.

**Figure 3:**
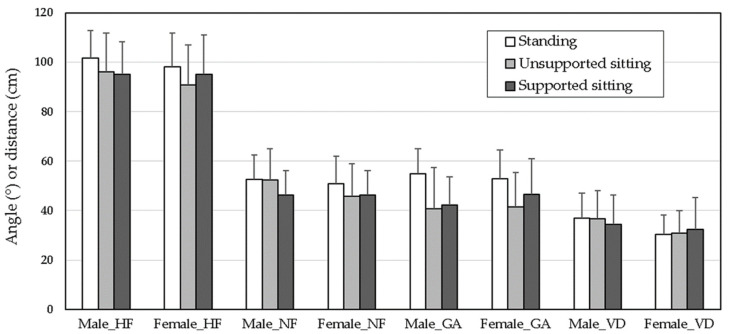
Comparison of measured angles and distances for various gender and posture combinations (HF, head flexion; NF, neck flexion; GA, gaze angle; VD, viewing distance).

Figure 3 compares the angles and distances measured for various gender and posture combinations. Both genders had greater HF and NF when operating a smartphone while standing than while sitting. Men had nearly identical NF while standing and while sitting unsupported, indicating that the load on the cervical spine is nearly equivalent in these positions. Gender differences in NF for the two sitting conditions can explain the significance of the interaction effect of gender and posture on NF (Table 3) and GA. Female users tended to have an erect neck–trunk posture, even during unsupported sitting, in contrast to the more casual, hunched posture of male users. The result agreed with the observation studied by Korakakis et al. [39], who found that females adopted more upright postures than males as they were requested to sit with their optimal postures; females sat with a more extended head, upper thoracic, lower thoracic, and lumbar spine, and with the pelvis more anteriorly tilted. In the current study, results showed that the postures of female users were generally more erect during unsupported sitting. However, NF was similar between genders (46.2° for men and 46.4° for women) during supported sitting because a backrest restricts body movement. Although static postures (especially sitting) tend to cause lower UT activation and higher fatigue levels and discomfort scores than dynamic postures [33], our findings revealed that NF was significantly lower among female users in either sitting position and among male users during supported sitting, indicating a lighter load on the neck [20].

This study has several limitations. Because a field study cannot be controlled in the same manner as a laboratory study, the anatomical markers may have been identified inaccurately despite satisfactory interobserver and intraobserver reliabilities. This can explain why the study was performed in summer, mainly because light clothing was conducive to more accurate identifying of the body markers. Whether the study results are applicable to winter when participants wear heavy clothing needs further clarification. Even though the unusual conditions had been excluded, there were still some confounding factors that were difficult to either control or evaluate in the study, for example, the type and weight backpacks, type of shoes, health status (any existing musculoskeletal problems or pain symptoms for the participants), lighting condition, time of day, standing with a backrest, and unnatural postures (people who lean heavily or asymmetrically). It should be noted that among the excluded participants (21%) there may be a preselecting effect that may have influenced the results. This field study therefore attempted to average across the factors through the relatively large sample size. Additionally, only static standing and sitting postures were observed in this study, it is unknown how long a participant was maintaining the posture when a photo was taken. Walking affects cervical spine movement [5] and is also worthy of further investigation. Finally, the usage of smartphone in public areas may not be representative of conditions at home; while at home, the phone could be used with head and/or arm supports. This field study was performed in Taipei, Taiwan; regional differences in posture of smartphone users may affect the generalizability of these results.

## 5. Conclusions

In contrast to previous studies that primarily used epidemiological approaches or simulated smartphone use experiments, this study performed field observations. The HF, NF, GA, and VD of 300 male and 300 female smartphone users in different postures in public areas were measured and analyzed. ANOVA results revealed that gender significantly affected HF, NF, and VD during smartphone use; that is, women had smaller HF and NF angles than men. In both the standing and sitting postures, the results indicate that smartphone use can pose a cervical spine hazard. During unsupported sitting, women tended to adopt an erect neck–trunk posture, whereas men tended to adopt a hunched and flexed neck posture. In all postures, women had a shorter VD than men (average difference 5.4 cm) and thus a greater risk of eye strain.

## Figures and Tables

**Table 1:** Correlation analysis (r) and mean absolute difference (MAD) of intraobserver reliability for the three measurers.

	Neck Flexion		Head Flexion		Gaze Angle		View Distance	
	r	MAD	r	MAD	r	MAD	r	MAD
Measurer 1	0.987	1.7°	0.960	2.0°	0.955	1.1°	0.970	2.4 cm
Measurer 2	0.990	3.1°	0.991	2.2°	0.993	2.3°	0.970	2.3 cm
Measurer 3	0.974	3.3°	0.952	3.2°	0.972	2.3°	0.964	2.6 cm

**Table 2:** Correlation analysis (r) of interobserver reliability between any two measurers and maximum mean absolute difference (MAD) among three measurers.

	Neck Flexion	Head Flexion	Gaze Angle	View Distance
Measurer 1 vs. 2	0.993	0.964	0.975	0.971
Measurer 2 vs. 3	0.980	0.934	0.981	0.936
Measurer 3 vs. 1	0.983	0.940	0.958	0.937
Maximum MAD	2.9°	3.1°	2.3°	2.2cm

**Table 3:** Two-way ANOVA results.

Sources	Variables	df	SS	MS	F	*p*	Power
Gender	Neck flexion	1	1063	1063	8.47	<0.01	0.828
	Head flexion	1	1293	1293	6.24	<0.05	0.703
	Gaze angle	1	154	154	0.88	0.349	0.155
	Visual distance	1	3384	3384	29.70	<0.001	1.000
Posture	Neck flexion	2	2917	1458	11.63	<0.001	0.994
	Head flexion	2	4418	2209	10.66	<0.001	0.989
	Gaze angle	2	17408	8704	49.85	<0.001	1.000
	Visual distance	2	6	3	0.03	0.972	0.054
Gender × Posture	Neck flexion	2	1134	567	4.52	<0.05	0.770
	Head flexion	2	712	356	1.72	0.180	0.361
	Gaze angle	2	1078	539	3.09	<0.05	0.595
	Visual distance	2	504	252	2.21	0.110	0.452

**Table 4:** Duncan grouping results for varying operating postures.

	Neck Flexion (°)			Head Flexion (°)			Gaze Angle (°)			View Distance (cm)		
Standing	51.7	(10.7)	A *	99.9	(12.5)	A	53.9	(10.9)	A	33.7	(9.7)	A
Unsupported sitting	49.1	(13.2)	B	93.5	(16.0)	B	41.2	(15.3)	C	33.9	(10.6)	A
Supported sitting	46.3	(9.9)	C	95.1	(14.6)	B	44.4	(13.2)	B	33.6	(12.4)	A

* Data (mean, with standard deviation in parentheses) with the same letter do not differ in the Duncan test.

**Table 5:** One-way ANOVA results for each gender.

Sources	Variables	df	SS	MS	F	*p*	Power
Males	Neck flexion	2	2573	1287	10.86	<0.001	0.990
	Head flexion	2	2574	1287	7.02	<0.001	0.926
	Gaze angle	2	12203	6102	35.65	<0.001	1.000
	View distance	2	286	143	1.13	0.323	0.249
Females	Neck flexion	2	1479	740	5.58	<0.01	0.854
	Head flexion	2	2557	1278	5.53	<0.01	0.851
	Gaze angle	2	6283	3142	17.66	<0.001	1.000
	View distance	2	225	113	1.10	0.333	0.243

**Table 6:** Duncan grouping results for various gender and posture combinations.

		Neck Flexion (°)			Head Flexion (°)			Gaze Angle (°)			View Distance (cm)		
	Standing	52.6	(10.0)	A *	101.7	(11.2)	A	55.0	(10.0)	A	36.9	(10.2)	A
Males	Unsupported sitting	52.3	(12.7)	A	96.1	(15.7)	B	40.8	16.8)	B	36.7	(11.4)	A
	Supported sitting	46.2	(9.8)	B	95.0	(13.3)	B	42.2	(11.5)	B	34.7	(11.9)	A
	Standing	50.9	(11.3)	A	98.0	(13.6)	A	52.8	(11.6)	A	30.5	(7.8)	A
Females	Unsupported sitting	45.9	(13.0)	B	90.9	(16.0)	AB	41.6	(13.7)	C	31.0	(8.9)	A
	Supported sitting	46.4	(10.0)	B	95.1	(15.9)	B	46.6	(14.5)	B	32.5	(12.8)	A

* Data (mean, with standard deviation in parentheses) with the same letter do not differ in the Duncan test.

## Data Availability

The data are available upon reasonable request to the corresponding author.
